# Genome-Wide Identification, Gene Structure and Expression Analysis of the MADS-Box Gene Family Indicate Their Function in the Development of Tobacco (*Nicotiana tabacum* L.)

**DOI:** 10.3390/ijms20205043

**Published:** 2019-10-11

**Authors:** Ge Bai, Da-Hai Yang, Peijian Cao, Heng Yao, Yihan Zhang, Xuejun Chen, Bingguang Xiao, Feng Li, Zhen-Yu Wang, Jun Yang, He Xie

**Affiliations:** 1 Tobacco Breeding and Biotechnology Research Center, Yunnan Academy of Tobacco Agricultural Sciences, Kunming 650021, China; 30431@163.com (G.B.); bioresearch2013@126.com (D.-H.Y.); yaohn@126.com (H.Y.); duckzyhsm@163.com (Y.Z.); cxjkm@163.com (X.C.); xiaobg@263.net (B.X.); 2 Key Laboratory of Tobacco Biotechnological Breeding, Kunming 650021, China; 3 National Tobacco Genetic Engineering Research Center, Kunming 650021, China; 4 China Tobacco Gene Research Centre, Zhengzhou Tobacco Research Institute, Zhengzhou 450001, China; PeijianCao@163.com (P.C.); likite2002@163.com (F.L.); 5 Hainan Key Laboratory for Sustainable Utilization of Tropical Bioresource, Institute of Tropical Agriculture and Forestry, Hainan University, Haikou, Hainan 570228, China; zywang@hainu.edu.cn

**Keywords:** *Nicotiana tabacum*, MADS-box, genome-wide analysis, floral organ, flower development

## Abstract

MADS-box genes play a pivotal role in various processes, including floral and seed development, controlling flowering time, regulation of fruits ripening, and respond to abiotic and biotic stressors in planta. Tobacco (*Nicotiana tabacum*) has been widely used as a model plant for analyzing the gene function, however, there has been less information on the regulation of flowering, and the associated genes. In the present study, a total of 168 NtMADS-box genes were identified from tobacco, and their phylogenetic relationship, chromosome locations, and gene structures were further analyzed. NtMADS-box genes can be clustered into four sub-families of Mα, Mγ, MIKC*, and MIKC^C^. A total of 111 NtMADS-box genes were distributed on 20 chromosomes, and 57 NtMADS-box genes were located on the unanchored scaffolds due to the complex and incomplete assembly of the tobacco genome. Expression profiles of NtMADS-box genes by microarray from 23 different tissues indicated that members in different NtMADS-box gene subfamilies might play specific roles in the growth and flower development, and the transcript levels of 24 NtMADS-box genes were confirmed by quantitative real-time PCR. Importantly, overexpressed *NtSOC1*/*NtMADS133* could promote early flowering and dwarfism in transgenic tobacco plants. Therefore, our findings provide insights on the characterization of NtMADS-box genes to further study their functions in plant development.

## 1. Introduction

The MADS-box gene family represents an important type of transcription factors, which is widely present in fungi, animals, and plants [1]. The name of MADS originates from the first characters of the *MINICHROMOSOME MAINTENANCE 1* (*MCM1*) gene of yeast [2], *AGMOUS* (*AG*) gene of *Arabidopsis thaliana* [3], *DEFICIENS* (*DEF*) gene of *Antirrhinum majus* [4], and *SERUM RESPONSE FACTOR* (*SRF*) gene of humans [5]. All the MADS-box proteins contain a conservative MADS domain at the N terminus consisting of 58–60 amino acid residues [6], and encode a transcription factor which can bind to the CArG box (CC-A-rich-GC) in the promoter region of their target genes [7].

The MADS-box gene family can be divided into two groups, type I and type II, based on the evolutionary lineage [8]. The type I MADS-box genes contain an SRF domain that exists in both plants and animals [9], while type II MADS-box genes encode MEF2-like proteins and MIKC-type proteins [9]. Moreover, the type II MADS-box genes have a less conserved K domain, while the structure of type I MADS-box genes does not contain this domain [8,10]. Based on the differences in gene structures, the type I MADS-box genes can be divided into three subfamilies (Mα, Mβ, and Mγ), while type II MADS-box genes can be divided into MIKC^C^ and MIKC* types [11]. To date, most of the well-known MADS genes are type II genes, such as floral homeotic genes from the ABCDE model belonging to the MIKC^C^ subfamily [12]. However, limited studies have focused on the function of type I MADS-box genes [13].

The identification of MADS-box gene family has been widely characterized in many plant species, including *Arabidopsis* [11], rice [14], maize [15], apple [16], Chinese jujube [17], bread wheat [18], tartary buckwheat [19], carnation [20], potato [21], cotton [22], grapevine [23,24], *Brassica rapa* [25], sesame [26], *Brachypodium distachyon* [27], soybean [28], tomato [29], and *Populus trichocarpa* [30]. The first systematic study of the MADS gene family was carried out in *Arabidopsis*, in which 107 MADS-box genes were identified, including five related subfamilies—25 Mα genes, 20 Mβ genes, 16 Mγ genes, six MIKC* genes, 39 MIKC^C^ genes—with an unassigned exception, *AGL33* [11]. Moreover, the *Arabidopsis* type II family contains 39 MIKC^C^ genes, which can be divided into 13 groups based on the results of phylogenetic analysis [23].

In planta, MADS-box genes have a pivotal role for various processes, including floral and seed development, controlling flowering time, and regulation of fruits ripening [3,31], however, studies on type I MADS-box genes have been less reported until now. It has been reported that the type I MADS-box genes might be involved in the process of plant reproduction, such as female gametophyte, embryo, as well as endosperm development [13]. For example, the *Arabidopsis AGL23* gene was involved in the development of seed and female gametophytes [13,32]. Importantly, the MIKC^C^ subfamily in type II contains a larger number of functional genes, particularly in flower morphogenesis [33]. In the MIKC^C^ subfamily, six subgroups of A, B, Bs, C, D, and E are important constituent genes of the ABCDE model for flower development [12,33]. In *Arabidopsis*, *AP1* from the A group acts as a meristem identity gene or as a floral organ identity gene, and promotes petal and sepal development [34]. *PISTILATA* (*PI*) and *APETALA3* (*AP3*) belong to the B group, and *Arabidopsis ap3* and *pi* mutants showed that petals were transformed into flower buds and stamens were replaced by carpel [35]. *AG*, *SHATTERPROOF1* (*SHP1*/*AGL1*), *SHP2* (*AGL5*), and *SEEDSTICK* (*STK* /*AGL11*) are in the C/D group [36]. Two reductant *Arabidopsis* genes, *SHATTERPROOF 1* and *SHATTERPROOF 2* (*SHP1* and *SHP2*), were identified for the ovule integument, and control the dehiscence zone of flower and fruit differentiation, where they are required to form lignin [37]. The E group includes *SEPALLATA* (*SEP1*, *SEP2*, *SEP3,* and *SEP4*), which has been involved in the regulation of flower development in *Arabidopsis* [38]. Furthermore, members of the MIKC^C^ family are involved in the regulation of plant flowering. For example, the *FLOWERING LOCUS C* (*FLC*) gene is mainly involved in the vernalization process in *Arabidopsis* and winter wheat [17,39,40,41,42]. *SUPPRESSOR OF OVEREXPRESSION OF CO 1* (*AtSOC1*) plays a critical role in vernalization and gibberellin signal integration for flowering [42]. The *SHORT VEGETATIVE PHASE* (*SVP*) gene is involved in the regulation of plant flowering, and overexpression of the *SVP* gene can promote plant flowering in *Arabidopsis* [34]. *MADS AFFECTING FLOWERING* (*MAF1*/*FLM*) regulates flowering time through altering the patterns of splicing under different temperature conditions [43]. *NtSVP*, a MADS-box gene from *Nicotiana* that belongs to the SVP clade, has been involved in pedicel development [26].

MADS-box genes can be engaged in the plant response to diverse abiotic and biotic stressors [21,22,27,44,45,46,47]. There are eight or six MADS-box genes in *Brassica rapa* that can be induced by drought or salt stress, respectively [48]. The expression of wheat *TaMADS2* was upregulated after infection by stripe rust fungus [49]. In rice, *OsMADS26* negatively regulates the resistance to pathogens and drought tolerance [44]. *OsMADS25* and *OsMADS27* respond to osmotic stress, while *OsMADS25*, *OsMADS27*, and *OsMADS57* can be slightly upregulated by nitrate [45,46]. *LsMADS55*, an *APETALA1* (*AP1*) homolog in lettuce, can respond to heat stress due to direct binding by a heat shock factor *LsHSFB2A-1* in the promoter region [47]. Moreover, some MADS-box genes from *Brachypodium distachyon* may be involved in stress responses under salt, drought, and low-temperature conditions [27]. Tobacco (*Nicotiana tabacum L.*) has been widely used as a model plant for analyzing gene function, however, there has been less information on the regulation of flowering and the associated genes. In the present study, a total of 168 NtMADS-box genes were identified from tobacco, and their phylogenetic relationship, chromosome locations, and gene structures were further analyzed. Moreover, the expression patterns of NtMADS-box genes were investigated based on the chip data from 23 different tissues, and 24 NtMADS-box genes were confirmed by quantitative real-time PCR (qPCR) assay. Importantly, overexpressed *NtSOC1*/*NtMADS133* could promote early flowering and dwarfism in the transgenic tobacco plants. Therefore, our findings provide valuable information on MADS-box genes to further study their functions in tobacco.

## 2. Results

### 2.1. Identification and Classification of MADS-Box Genes in Tobacco

Local BLAST and HMM analyses based on the China Tobacco Genome Database (*N. tabacum*) were performed, and genes such as pseudogenes, premature stop codons genes, or without complete MADS domain were removed. Finally, a total of 168 remaining genes were considered putative tobacco MADS-box genes, which were named from *NtMADS1* to *NtMADS168* (Supplementary Table S1). Interestingly, there was a large number of MADS-box genes in tobacco—more than soybean (163), apples (142), *Arabidopsis* (107), poplar (101), sorghum (76), rice (75), and maize (75). The coding sequences (CDS) lengths of NtMADS-box genes ranged from 183 to 1404 bp, which encoded variable numbers of amino acids (60 to 467). The molecular weights of deduced proteins were 6665.31 to 53,143.54 Da, with a pI of 3.7675 to 11.0189 (Supplementary Table S1).

**Figure 1:**
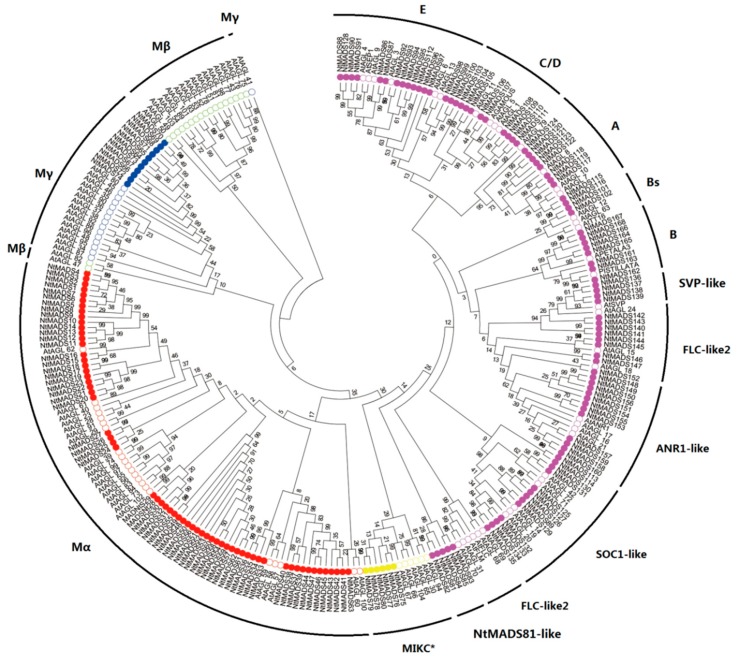
Phylogenetic analysis of NtMADS-box proteins from *Arabidopsis* and cultivated tobacco. A total of 107 AtMADS-box proteins from *Arabidopsis* (*Arabidopsis thaliana*) and 168 NtMADS-box proteins from cultivated tobacco were used to generate the unrooted neighbor-joining (NJ) tree with 1000 bootstrap replicates. The MADS-box proteins are classified into five subfamilies (marked as Mα, Mβ, Mγ, MIKC*, and MIKC^C^), and distinguished by different colors: NtMADS-box proteins are labeled in a solid circle, and AtMADS-box proteins are labeled in a hollow circle.

In order to understand the evolutional relationship of tobacco NtMADS-box genes, an unrooted phylogenetic tree was created based on the MADS protein sequences, including 168 tobacco NtMADS-box, 107 *Arabidopsis* AtMADS-box [11], and 107 tomato SlMADS-box (Figure 1 and Supplementary Figure S1) [29]. Consistent with previous studies [11], the phylogenetic analysis results showed that tobacco NtMADS-box genes can be divided into two types, type I and type II (Figure 1). Tobacco NtMADS-box genes can be further clustered into four sub-families of Mα, Mγ, MIKC*, and MIKC^C^, with a lack of Mß, while there were five sub-families in *Arabidopsis* and tomato [11,29]. Among the tobacco type I genes, there are 63 genes (*NtMADS1* to *NtMADS63*) in the Mα group, and 11 genes (*NtMADS64* to *NtMADS74*) in the Mγ group, while in the type II genes, there are six genes (*NtMADS75* to *NtMADS80*) in the MIKC* group, and 88 genes (*NtMADS81* to *NtMADS168*) in the MIKC^C^ group. Actually, most members within the Mα, Mγ, and MIKC* subfamilies had a close relationship in their particular species, while for members in the MIKC^C^ subfamilies, all genes were uniformly distributed among *Arabidopsis*, tomato, and tobacco. However, two *Arabidopsis* genes, *AtAGL61* and *AtAGL62*, were closer to those members of tobacco but distant from those members of *Arabidopsis*. To study selection pressures among duplicated NtMADS-box genes, the substitution ratios of non-synonymous (Ka) to synonymous (Ks) mutations (Ka/Ks) were calculated for the 111 gene pairs (Supplementary Table S2). The Ka/Ks values of most gene pairs were less than 1, suggesting that these duplicated NtMADS-box gene pairs evolved under purifying selection in *N. tabacum*.

The tobacco MIKC^C^ family can be divided into 11 clades, including A, B, Bs, C, D, E, SVP-like, FLC-like, ANR1-like, SOC-like, and NtMADS81-like (Figure 1). The NtMADS81-like clade, including five members (*NtMADS81* to *NtMADS85*), was identified as a distinct group that is specific to tobacco. Moreover, the FLC-like clade can be divided into two independent clades, named FLC-like1 and FLC-like2. Subsequently, the gene distribution of the MIKC^C^ gene family is more uniform. In the MIKC^C^ subfamily, most of the *Arabidopsis* subfamily MADS-box genes had corresponding the tobacco MADS-box genes, and the quantitative ratio was approximately 1:3 to 1:4 (Figure 1).

### 2.2. Structure of MADS-Box Genes in Tobacco

**Figure 2:**
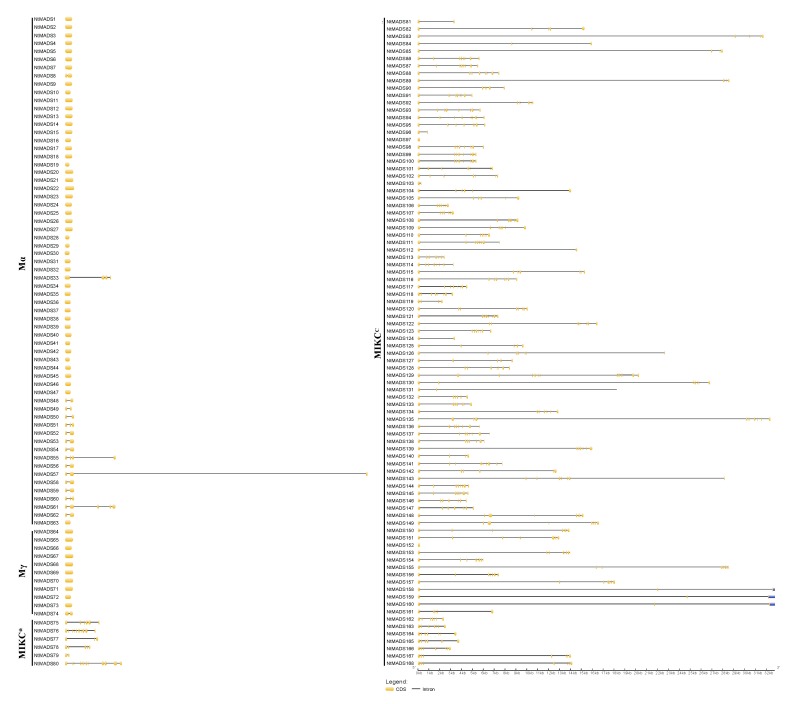
Gene structure of the NtMADS-box gene family in tobacco. Exon–intron analyses of identical tobacco NtMADS-box genes were performed with GSDS 2.0. Exons and introns are represented by black rectangle and black lines, respectively. The lengths of exons and introns for each tobacco NtMADS-box gene are shown proportionally.

To further obtain information on the gene structures of NtMADS-box, the CDS sequences and genomic sequences of NtMADS-box genes were compared. The results show that NtMADS-box genes contain conserved gene structures (Figure 2). The type II NtMADS-box genes had more introns than the type I MADS-box genes, and the lengths of introns in the type II NtMADS-box genes were longer than those in the type I NtMADS-box genes (Figure 2). Most members of type I NtMADS-box genes did not have intron, while only a few had one or two introns (Figure 2). Surprisingly, *NtMADS33* and *NtMADS55* had three introns, and NtMADS61 had four introns. It is interesting that introns of the type I MADS-box genes were usually shorter than 1 kb, except for *NtMADS57*, whose second intron was more than 27 Kb (Figure 2). The number of exons in the MIKC^C^ family gene was more than that of other NtMADS-box subfamilies, and most MIKC^C^ family genes contained more than four exons, except for *NtMADS89*, *NtMADS96*, *NtMADS97*, *NtMADS103*, *NtMADS112*, *NtMADS119*, *NtMADS124*, *NtMADS140*, *NtMADS159,* and *NtMADS160* (Figure 2). Furthermore, the MIKC^C^ family genes usually had longer introns, where most of them were more than 5 kb, and some genes had more than 20 kb in introns, such as *NtMADS89* (Figure 2).

### 2.3. Motif Analysis of MADS-Box Genes in Tobacco

**Figure 3:**
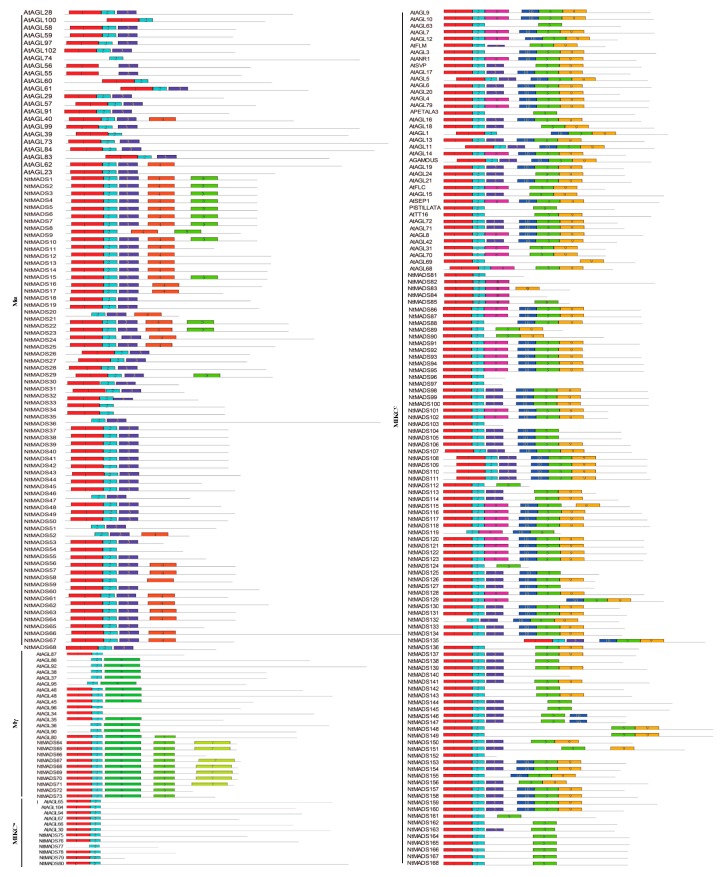
Conserved motifs of MADS-box genes in tobacco and *Arabidopsis* were predicted by MEME. Grey lines represent the non-conserved sequences, and ten conserved motifs are indicated by different colors with numbered boxes.

To identify motifs in the NtMADS-box gene family, motifs of MADS-box proteins in tobacco and *Arabidopsis* were analyzed by MEME software, and then the obtained 10 motifs were blasted and functionally annotated by HMMER. Motif 1 and motif 2 encoded the SRF domain, which is the most conserved domain, with 48 amino acids from SRF-type MADS transcription factors (Figure 3), and most MADS-box members from *Arabidopsis* and tobacco had these two motifs, while only some members had one of these two motifs, such as eight *Arabidopsis* members (AtAGL36, AtAGL37, AtAGL38, AtAGL74, AtAGL80, AtAGL86, AtAGL92, and AtAGL95) and eight tobacco members (NtMADS19, NtMADS33, NtMADS43, NtMADS47, NtMADS48, NtMADS77, NtMADS119, and NtMADS132), which had only motif 2, while only two genes from the *Arabidopsis* Mα family (AtAGL55 and AtAGL56) had motif 1. Motif 3 was widely present in the Mα family of *Arabidopsis* and tobacco (Figure 3). However, four *Arabidopsis* members (AtAGL39, AtAGL60, AtAGL74, and AtAGL100) and four tobacco members (NtMADS31, NtMADS32, NtMADS50, and NtMADS54) did not have motif 3.

Motif 4 was specifically distributed in the 31 members from tobacco, and in the two *Arabidopsis* members from the Mα family (AtAGL40 and AtAGL62), while genes from other subfamilies did not have motif 4 in *Arabidopsis* (Figure 3). Three motifs, including motif 5, motif 9, and motif 10, could form the K domain. Motif 5 was distributed in most members from the MIKC^C^ subfamily of *Arabidopsis* and tobacco, and a few members from tobacco were in the Mα and Mγ subfamily. In tobacco, 13 members (NtMADS1 to NtMADS9, NtMADS14, NtMADS20, NtMADS21, and NtMADS27) from the Mα subfamily and 10 members (NtMADS64 to NtMADS73) from the Mγ subfamily had motif 5, but there was none of motif 5 in the *Arabidopsis* Mα, Mγ, or MIKC* subfamilies, except for AtAGL80 (Figure 3). Moreover, six members from the MIKC^C^ subfamily lacked motif 5, including AtAGL69, NtMADS96, NtMADS97, NtMADS103, NtMADS140, and NtMADS152. Most members from the MIKC^C^ subfamily had motif 9, but three *Arabidopsis* members (AtAGL63, AP3, and PISTAL) and 25 members from tobacco lacked motif 9. Among the MIKC* subfamily, only NtMADS83 contained motif 9 (Figure 3). Motif 10 was confined to the MIKC^C^ family. Among them, 14 *Arabidopsis* members and 33 members from tobacco lacked motif 10 (Figure 3).

It was noted that all genes from the *Arabidopsis* MIKC^C^ subfamily had K domain motifs, while tobacco *NtMADS96*, *NtMADS97*, *NtMADS103*, *NtMADS140*, and *NtMADS152* did not have the K domain (Figure 3). Most of the genes from the Mγ subfamily had motif 6 compared to other subfamily genes in *Arabidopsis* and tobacco, while only *Arabidopsis AtAGL34, AtAGL87*, and *AtAGL96* lacked this motif. Motif 7 was only present in the tobacco Mγ subfamily—11 members have motif 7, while only three genes lacked this motif, *NtMADS66*, *NtMADS72*, and *NtMADS73*. Motif 8 was present in the MIKC-type genes, including four members from the tobacco MIKC* subfamily, 16 members from the *Arabidopsis* MIKC^C^ subfamily, and 18 members from the tobacco MIKC^C^ subfamily (Figure 3).

### 2.4. Location of MADS-Box Genes in Tobacco

**Figure 4:**
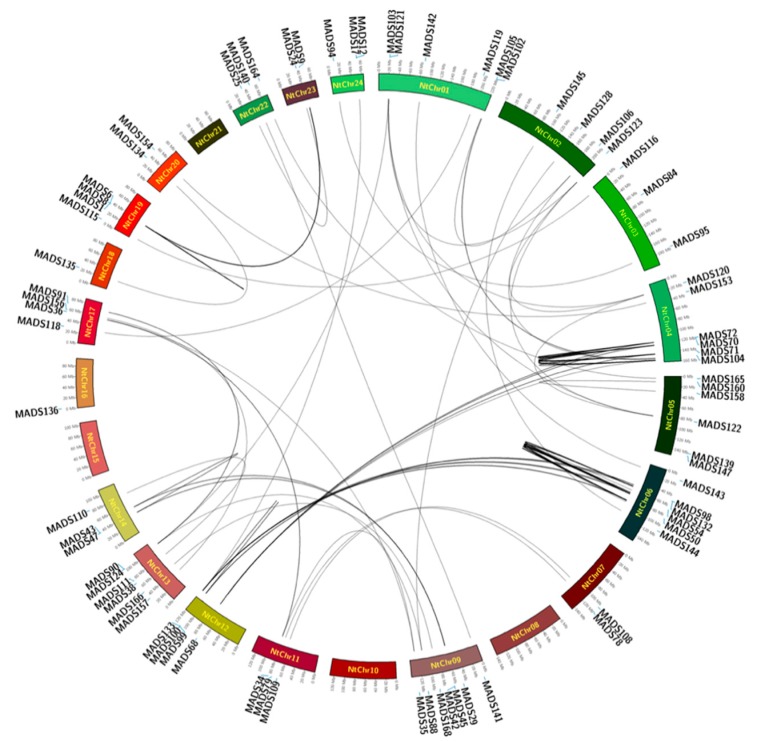
Collinear analysis of the NtMADS-box gene family in tobacco. The annulus represents the tobacco chromosomes, and the scale on the annulus is labeled in megabases (Mb). Homologous genes are linked by the lines. The figure was generated and modified using the Circos program.

A total of 111 NtMADS-box genes were distributed on the 20 chromosomes, separately. Furthermore, none of genes were located on chromosomes 8, 10, 15, or 21 (Figure 4). A total of 57 NtMADS-box genes could not be distributed on the tobacco chromosomes, but were located on the unanchored scaffolds (Figure 4). This might be due to the complex and incomplete assembly of the tobacco genome. Meanwhile, the number of NtMADS-box genes that were distributed on each tobacco chromosome ranged from 1 to 22. There was only one NtMADS-box gene located on Nt-chr16 and Nt-chr18 (Figure 4). The greatest number of NtMADS-box genes was mapped to Nt-chr6 (22 genes), followed by Nt-chr4 (12 genes). Less than 10 genes were located on other chromosomes (Figure 4). It is well known that gene duplication plays an important role in gene functional differentiation [50]. There were six tandem duplications: *NtMADS70*, *NtMADS72,* and *NtMADS74*; *NtMADS66* and *NtMADS67*; *NtMADS49*, *NtMADS59,* and *NtMADS62*; *NtMADS26* and *NtMADS27*; *NtMADS19* and *NtMADS22*; and *NtMADS1* to *NtMADS8*. Moreover, a gene cluster can be formed among *NtMADS70*, *NtMADS72,* and *NtMADS74*; *NtMADS49*, *NtMADS59,* and *NtMADS62*; and *NtMADS1* to *NtMADS8* (Figure 4).

### 2.5. Expression Patterns of MADS-Box Genes in Tobacco

**Figure 5:**
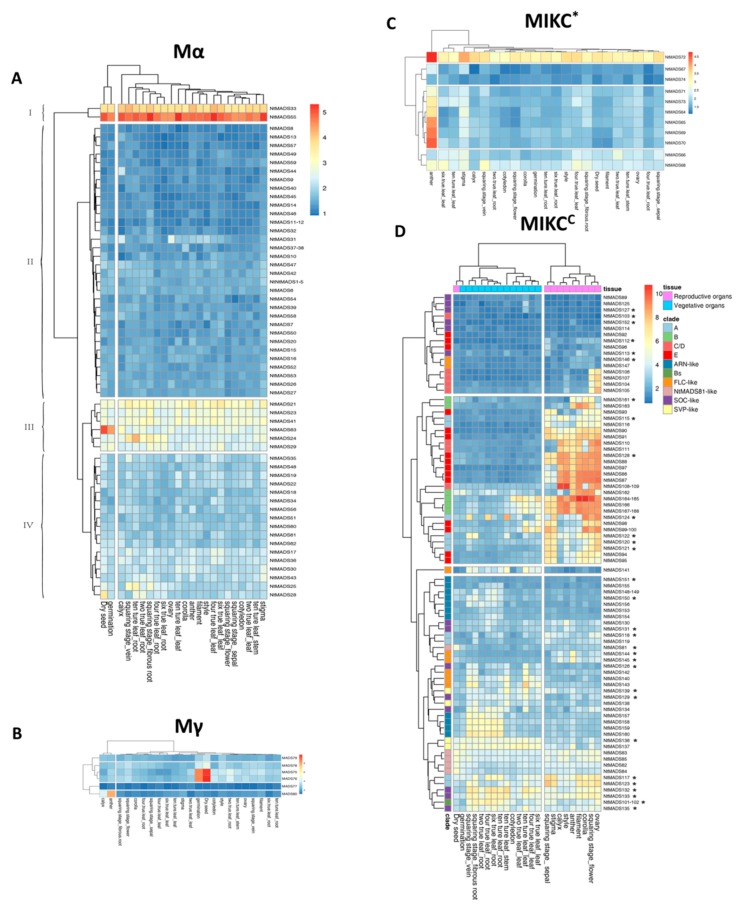
Expression profiles of 168 NtMADS-box genes in tissues at different developmental stages. The relative transcript abundances of 168 *NtMADS-box* were examined via microarray and visualized as a heatmap. The expression profiles of NtMADS-box genes in the 23 different samples, including dry seeds, germination seeds, cotyledons, leaves from two-true leaf stage (labeled as two true leaf_leaf), roots from two-true leaf stage (two true leaf_root), leaves from four-true leaf stage (four true leaf_leaf), roots from four-true leaf stage (four true leaf_root), leaves from six-true leaf stage (six true leaf_leaf), roots from six-true leaf stage (six true leaf_root), leaves from ten-true leaf stage (ten ture leaf_leaf), roots from ten-true leaf stage (ten ture leaf_root), and squaring stage (sepal, fibrous root, and flower), vein, ovary, filament, style, corolla, calyx, stigma, and anther. The X axis is the samples in tissues at different developmental stages. The color scale represents Log2 expression values. The symbol of the star in the MIKC^C^ subfamily represents selected genes for confirming the gene expression by qPCR. Three independent biological experiments with four individual plants were collected for RNA extraction.

To further elucidate the function of the tobacco MADS-box gene family, gene chips of 23 tobacco tissues were used to detect the expression patterns of NtMADS-box genes, and three independent chips were performed for each tissue to obtain accurate expression levels. Due to the large number of members of the NtMADS-box family, their transcript data were analyzed based on each subfamily, and expression was shown as Log2 fold (Figure 5, Supplementary Table S3). The expression patterns of the Mα subfamily could be divided into four clades (I, II, III, and IV) as shown in Figure 5. Clade I includes two genes, *NtMADS33* and *NtMADS55*, whose expression levels were between 3.8 and 5. The expression level of *NtMADS55* was higher than that of *NtMADS33* in almost detected tissues. Clade II and IV contain 55 genes, and all genes had an expression level below 3, and the expression levels of clade II genes were lower than those of clade IV genes (Figure 5). Clade III has six genes, *NtMADS21*, *NtMADS23*, *NtMADS24*, *NtMADS29*, *NtMADS41,* and *NtMADS63*, whose expression levels ranged between 2 and 3 in most tissues. Particularly, the *NtMADS63* gene showed the highest expression in dry seeds and germination seeds, with levels of 5.07 and 4.43, respectively. The Mγ subfamily showed similar expression patterns to the Mα subfamily (Figure 5). Among them, the expression level of *NtMADS72* was more than 3, while other Mγ genes showed expression levels below 2.5. Interestingly, eight genes from this subfamily (*NtMADS64*, *NtMADS65*, *NtMADS68*, *NtMADS69*, *NtMADS70*, *NtMADS71*, *NtMADS72,* and *NtMADS73*) exhibited higher expression levels in anther, of more than 2.7. Among the MIKC* subfamily, the expression levels of *NtMADS77* and *NtMADS80* were lower under 2. *NtMADS75*, *NtMADS76*, *NtMADS78,* and *NtMADS79* widely had expression levels of 4 to 5 in most tested tissues (Figure 5). However, *NtMADS80* showed specific expression patterns, with the highest levels in anther, of up to 7.8, and in calyx, as high as 4.26. At the same time, both *NtMADS75* and *NtMADS76* genes had higher expressions in day seeds and germination seeds, which were more than 8 (Figure 5). The expression levels of most MIKC^C^ members were more than those of genes from other subfamilies. The expression levels of A subfamily members, except *NtMADS118* and *NtMADS119*, were more than 6 in sepal, stigma, filament, corolla, and ovary, while the expression levels of *NtMADS118* and *NtMADS119* were less than 4 in those tissue (Figure 5). For the A subfamily, the expression levels of *NtMADS161* to *NtMADS163* showed 5.6 in anther, and 4.8 in filament, while the expression levels of other members in this subfamily were more than 6 in anther, filament, and corolla. Notably, the expression levels of *NtMADS164* to *NtMADS168* were more than 7 in all flower organs. The expression levels of the *NtMADS101* to *NtMADS102* genes from the Bs subfamily showed more than 5 in roots and ovary (Figure 5).

Among the C/D subfamily, the *NtMADS104* to *NtMADS107* genes were abundantly expressed in the ovary, with levels more than 7, while the expression levels of *NtMADS108* to *NtMADS111* were more than 5 in sepal, style, anther, filament, and ovary (Figure 5). Among the E subfamily, *NtMADS88*, *NtMADS91,* and *NtMADS128* had expression levels of less than 6 in calyx, anther, and style, while the transcripts of other members were expressed more than 6 in sepal, stigma, calyx, style, anther, filament, and ovary. The *NtMADS86*, *NtMADS87,* and *NtMADS91* genes showed higher expressions, with more than 5 in calyx, style, anther, filament, corolla, and ovary (Figure 5). The expression levels of *NtMADS98* to *NtMADS100* were more than 6 in sepal, stigma, corolla, and ovary. *NtMADS94* and *NtMADS95* were expressed in sepal, stigma, filament, corolla, and ovary with levels of more than 5. However, the expression levels of *NtMADS92*, *NtMADS96,* and *NtMADS112* were less than 4 in all tested tissues (Figure 5). Among the ANR-like subfamily, the *NtMADS157* to *NtMADS160* genes showed expression levels higher than 5 in roots, while the expression levels for *NtMADS148* to *NtMADS150* and *NtMADS153* to *NtMADS156* were lower in roots, between 2 and 5, and the expression level of *NtMADS151* was less than 2 (Figure 5). Among the FLC-like subfamily, the expression levels of the *NtMADS140* to *NtMADS143* genes were 3 to 6 in roots and leaves, and *NtMADS144* and *NtMADS145* were expressed between 3 and 6 in stigma, calyx, style, anther, filament, and ovary, while the expression levels of the *NtMADS146* and *NtMADS147* genes were less than 3 in all the tissues (Figure 5).

All members of the NtMADS81-like subfamily, except *NtMADS81*, were expressed at 3 to 5 in all tissues (Figure 5). *NtMADS81* showed the highest expression level of 5.2 in dry seeds and less than 4 in other tissues. Among the SOC-like subfamily, *NtMADS132*, *NtMADS133,* and *NtMADS135* showed expression in all tissues with levels of 4 to 7.5. *NtMADS135* showed less expression in flower organs than *NtMADS132* and *NtMADS133*. The expression levels of *NtMADS126* and *NtMADS129* were 4 to 6 in roots and leaves, and less than 4 in flower organs (Figure 5). Among the SVP-like subfamily, the *NtMADS136* and *NtMADS137* genes had higher expression levels (6) in roots and leaves, while the expression levels of the *NtMADS138* and *NtMADS139* genes were lower in roots and leaves than those of *NtMADS136* and *NtMADS137* (Figure 5).

**Figure 6:**
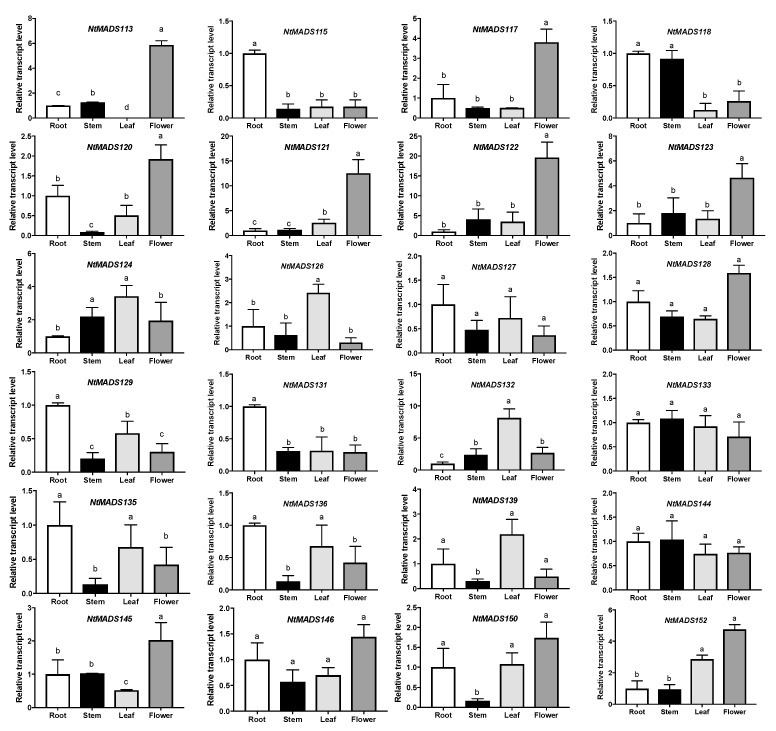
Expression profiles of 24 selected NtMADS-box genes in tobacco. The relative transcript abundances of 24 selected NtMADS-box genes were examined via qPCR and visualized as a histogram. The tobacco flowers (120 day old plants) and 6–7 week old seedlings grown in the soil were collected. Three independent biological experiments with four individual plants were collected for RNA extraction and qPCR analysis. 26S was used as an internal control. Error bars represent the SD (*n* = 3). Different letters a,b,c above the bars indicate a significant difference (*p* < 0.05), as obtained by one-way ANOVA and the LSD test.

It is well known that NtMADS-box genes in the MIKC^C^ subfamily are involved in the development and control of flowers [10]. To further confirm the expression patterns of NtMADS-box genes in diverse organs in tobacco, and predict their potential role in the development and regulation of flowering time, 30 genes belonging to 11 clades in the MIKC^C^ subfamily were random selected (Figure 5). However, the expression levels of six genes were varied and lower, that we could not analyze further by qPCR in the tobacco plants. Therefore, 24 genes were then analyzed for indicated tissue. It was found that several NtMADS-box genes, *NtMADS113*, *NtMADS117*, *NtMADS120*, *NtMADS121*, *NtMADS122*, *NtMADS123*, *NtMADS128*, *NtMADS145*, and *NtMADS152,* had the highest expression levels in flowers than those in other organs (Figure 6), while some NtMADS-box genes, for example, *NtMADS115*, *NtMADS129*, and *NtMADS131,* had the highest expression levels in root, and *NtMADS124*, *NtMADS126*, *NtMADS132,* and *NtMADS139* had the highest expression levels in the leaf when compared with other organs (Figure 6). *NtMADS118* had higher expression levels in the root and stem, and *NtMADS135* and *NtMADS136* had higher expression levels in the root and leaf when compared with other organs (Figure 6). However, the expression levels of some genes, such as *NtMADS113*, *NtMADS115, NtMADS118*, *NtMADS124,* and *NtMADS131,* were not consistent with chips data, which might be due to different samples and growth stages.

### 2.6. Identification of NtSOC1 in Regulating the Flower Time and Development in Tobacco

It has been suggested that SOC1 participates in the positive regulatory process of flowering time in multiple species [51,52,53,54], and overexpressed SOC1 could cause early flowering in transgenic plants [51,52,53,54]. However, the potential role of NtSOC1 in controlling flowering is not known in tobacco. GUS staining demonstrated that the *AtSOC1* gene was widely expressed in diverse tissues, except for mature seeds [55]. According to the evolutional relationship of NtMADS-box genes, there are 14 SOC1-like genes. Among them, *NtMADS89*, *NtMADS113*, *NtMADS114*, *NtMADS125*, *NtMADS127*, *NtMADS130*, *NtMADS131,* and *NtMADS152* showed lower expression levels, while *NtMADS126*, *NtMADS129,* and *NtMADS134* were highly expressed in vegetative tissues, and lower in reproductive tissues. However, *NtMADS133* showed constitutive expression patterns in the tissues (Figure 6), that was similar to *AtSOC1* gene expression in previous study [51].

**Figure 7:**
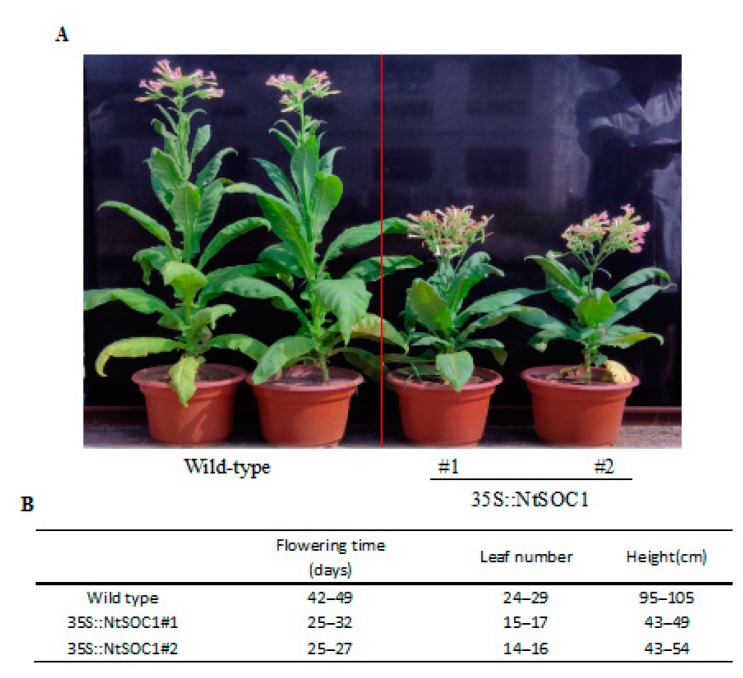
Phenotypes of *NtSOC1*/*NtMADS133* overexpressed transgenic lines in tobacco. (**A**) Growth of wild-type and two *NtSOC1* overexpressed lines. Seedlings were grown in soil for 100 days. Representative plants were photographed. (**B**) Quantitative analysis of wild-type and two *NtSOC1* overexpressed lines on the height and flower time. Three independent biological experiments with 12 individual plants per transgenic lines were calculated.

To dissect the function of *NtSOC1* genes, the *NtMADS133* gene was chosen and overexpressed in tobacco. It was shown that overexpressed *NtMADS133* resulted in early flowering, decreased leaf number, and dwarfism in transgenic plants (Figure 7). This took 58 days from transplanting to flowering for non-transgenic plants, but only 38 days for the transgenic plants (Figure 7B). Furthermore, there were 26 leaves when non-transgenic plants flowered, in contrast with overexpressed lines, which showed significantly lower leaf numbers (on average, 15.3) (Figure 7B). The height of tobacco plants was reduced from 97 cm for non-transgenic plants to 47.2 cm for overexpressed transgenic plants (Figure 7B).

## 3. Discussion

The MADS-box gene family is important for plant growth and development, which has been widely studied in many crops [15,16,23,28,30,44]. In this study, a total of 168 tobacco NtMADS-box genes were identified, which is more than for most plants, but less than in bread wheat (180) [18]. *N. tabacum* has more MADS-box genes mainly because *N. tabacum* is allotetraploid, with genomes as large as 4.5 Gb, which is caused by the hybrid between *Nicotiana sylvestris* and *Nicotiana tomentosiformis* in the process of evolution [56].

Like other plants, tobacco also has more Mα and MIKC^C^ genes than Mγ and MIKC* genes (Figure 1). The Mβ gene family is missing in the tobacco genome, which is consistent with bread wheat [18]. None of Mβ gene family members have been identified in tobacco, probably due to the incomplete tobacco genome, or members of Mβ gene family have been lost in tobacco during evolution. The distribution of NtMADS-box subfamily genes in tobacco is consistent with the distribution of MADS-box subfamilies in most plants, indicating that the plant MADS-box gene family is evolutionarily conserved [11,16,28]. Many studies reported that the MIKC^C^ gene family plays a central role in flower development and flowering time regulation [1,34,35,36,37,42,57,58]. It is interesting that there are 83 MIKC^C^ in tobacco, indicating that there may be a complex flowering regulation mechanism in tobacco (Figure 1). Moreover, a reduction in the numbers of subfamilies, such as Mβ, Mγ, and MIKC*, indicates that these family genes may gradually disappear during evolution.

Gene structure is usually conservative in the evolution process [59,60]. It was found that the gene structure of the tobacco NtMADS-box gene family is the same as that of *Arabidopsis* MADS-box genes, in which the type I gene family has fewer introns, and the type II gene family gene has more introns. These results indicate that the MADS-box gene family is conserved during evolution. Furthermore, the type II genes in tobacco have more intron numbers than those of type I genes, suggesting that type II genes are more conservative than type I genes. Similarly, the type II genes might have more important biological functions than type I in tobacco, which is consistent with previous studies on *Arabidopsis*. For example, MIKC^C^ genes in the type II gene family are involved in regulating flower development, flowering time, and root development, while type I genes are not. Previous studies showed that evolutionarily conserved genes have a greater intronic burden, and a positive association between the level of evolutionary conservation and the size of intronic region of a gene for eukaryotic genes [61]. Gene expression with a small total intron size (less than 1 kb) was relatively lower and increased markedly until the total intron size reached 5 kb. For genes with total intron lengths greater than 5 kb, there was a negative association with expression. Moreover, intron length may affect many processes in addition to the switch between intron definition versus exon definition, and novel exons are more frequently present in long introns than in short introns [62,63,64]. It was suggested that splicing regulatory sequences increased density with increasing intron length (less than 1.5 kb), while increasing intron lengths (more than 1.5 kb) are associated with increased splice site strength [65]. Therefore, various intron patterns between type I and type II genes may have evolutionary conservation on the expression or splicing regulatory in tobacco.

In planta, motif 1 and motif 2 encode the MADS SRF-type transcription factor domain, which is the most conserved among the NtMADS-box gene family. Moreover, motif 5, motif 9, and motif 10 can combine together to constitute the K box domain, which is the second conserved domain in the NtMADS-box gene family (Figure 3). Usually, the motif of the K box domain only exists in the MIKC^C^ family [11]. Contrasting with *Arabidopsis*, motif 5 of the K box domain is also present in other non-MIKC^C^ families in tobacco. Currently, only ZjMADS51 of Chinese jujube has been found in the non-MIKC* families that contain a K box motif [17]. These results suggest that the diversity of MADS-box genes in tobacco may be more abundant, and other tobacco subfamilies containing a K box domain motif require further study.

The MIKC^C^ subfamily genes in the MADS-box gene family are involved in the regulation of floral development, flowering time, and root development; therefore, the gene expressions of most members of this subfamily usually exhibit a strong tissue specificity [10,11,12,66,67], and accordingly, their specific tissue expression is probably related to gene function. For example, *AGL15* plays a potential role in embryogenesis, and the overexpression of *AGL15* promotes the production of secondary embryos [68]. An important repressor of floral transition, *FLC*, allows the plant to flower, which is controlled by vernalization, and *FLC* expression is downregulated by epigenetic chromatin regulators and possibly by long non-coding RNAs. Another MIKC^C^-type floral repressor, *SVP*, interacts with *FLC* [69], and *FLC* and *SVP* repress the expression of *FT* and other genes that initiate floral transition [69,70,71]. *AGL17* clade gene *ARABIDOPSIS NITRATE REGULATED 1* (*ANR1*), which functions in nutrient response, controls lateral root elongation in response to nitrate [72,73]. The auxin-dependent cell-cycle is controlled by *XAANTAL1* (*XAL1*; *AGL12*), which affects root growth and flowering time [74]. TM3/SOC1 clade genes are also expressed in the root, and control floral transition in shoots [75]. In Gerbera, the SEP1 orthologue *GERBERA REGULATOR OF CAPITULUM DEVELOPMENT 2* (*GRCD2*) functions in inflorescence determinacy [76] and controls inflorescence architecture [77]. The *VEG1* gene, which is an AGL79-like gene (SQUA subfamily), has a role in controlling inflorescence architecture [78]. The expression of the MIKC^C^ subfamily gene in tobacco is conserved and can be divided into three major categories (Figure 4). The first category is mainly expressed in floral organs, including A, B, Bs, C/D, and E clade. The second type is mainly expressed in leaves, such as SVP-like, SOC-like, and FLC-like clade. The third category is mainly expressed in roots, such as ANR-like. Therefore, gene function might be predicted based on the characteristics of gene expression in the MIKC^C^ subfamily. The expression patterns of NtMADS-box genes were investigated in 23 tobacco tissues, which was the most comprehensive expression study for MADS-box genes in tobacco, and showed strongly tissue-specific expression. Therefore, tissue-specific analysis of gene expression in the NtMADS-box gene family may provide insight on the functional characteristic of genes that are involved in the regulation of tobacco root development, leaf development, flower development, and regulation of flowering time.

In *Arabidopsis*, the *AtSOC1* gene has been considered an important regulator for prompting early flowering [51]. However, less information was reported on the functional analysis of the *NtSOC1* gene in tobacco, particularly in its regulation of flowering. In the present study, we identified a putative *NtSOC1* gene, *NtMADS133*, which showed similar expression patterns to *AtSOC1* (Figure 6) [55]. It has been reported that transgenic *Arabidopsis* with overexpression of the *AtSOC1* gene showed changed phenotypes, including fewer rosette leaves and early flowering [51]. *AtSOC1* expression is also regulated by *FLC* and by the gibberellin-dependent signaling pathway, indicating that *AtSOC1* has been identified as one of floral pathway integrators [79]. However, individual SOC1-like genes may play different roles in different species, even in the same species, such as *OsMADS50* and *OsMADS56*, which antagonistically function in the regulation of flowering by controlling the expression of *OsLFL1* and *Ehd1* [80]. The ectopic expression of *OsMADS50* promotes early flowering, but overexpression of *OsMADS56* leads to delay flowering [80]. Most studies of early-flowering genes, including *FT*, exhibited early flowering and dwarfed growth in the transformed plants [81,82]. Moreover, overexpressing the petunia SOC1-like gene *FBP21* in tobacco showed dwarfed growth and earlier flowering [83]. The ectopic expression of a SOC1 homolog from *Phyllostachys violascens* changes the flowering time and identity of dwarfism in *Arabidopsis thaliana* [84]. Consistent with a previous study, overexpressing *NtSOC1*/*NtMADS133* in tobacco showed early flowering and dwarf phenotype (Figure 7), suggesting their conserved functions on the development of growth and flowers in planta.

## 4. Materials and Methods

### 4.1. Plant Materials and Growth Conditions

*N. tabacum L*. was used in analyzing the expression profiles of NtMADS-box genes. Seeds of tobacco cv. Yunyan87 were obtained from the Yunnan Academy of Tobacco Agricultural Sciences (Yunnan, China) [66]. Seeds were surface-sterilized in 40% bleach solution for 10 min, followed by three washes in sterile distilled water, and directly sowed into the soil in pots. Young tobacco seedlings were grown in a plant growth chamber with a 16-h light/8-h dark photoperiod under continuous white light (∼75 mol m^−2^ s^−1^) at 28 °C—day/ 23 °C—night. All plants were kept well-watered after sowing.

Tobacco samples were collected from plants in the field and flash-frozen in liquid nitrogen. Field management was performed according to normal agricultural practices. The collected samples included 23 different tissues, including dry seeds, germination seeds, cotyledons, leaves from the two-true leaf stage (labeled as two true leaf_leaf), roots from the two-true leaf stage (two true leaf_root), leaves from the four-true leaf stage (four true leaf_leaf), roots from the four-true leaf stage (four true leaf_root), leaves from the six-true leaf stage (six true leaf_leaf), roots from the six-true leaf stage (six true leaf_root), leaves from the ten-true leaf stage (ten ture leaf_leaf), roots from the ten-true leaf stage (ten ture leaf_root), and the squaring stage (sepal, fibrous root, and flower), vein, ovary, filament, style, corolla, calyx, stigma, and anther. Each tissue sample had three biological replicates for further experiments.

### 4.2. Phylogenetic and Gene Structure Analyses

The NtMADS-box gene sequences were obtained from the China tobacco genome database V2.0 and were predicted by HMMER [85]. The sequences of AtMADS-box were collected from the NCBI GeneBank database. NtMADS-box and AtMADS-box were aligned using ClustalW [86], and an unrooted phylogenetic tree was generated using MEGA 7.0 (https://www.megasoftware.net/) by the neighbor-joining method, with 1000 replicates of bootstrap analysis [87].

Each NtMADS cDNA sequence was aligned with its corresponding genomic DNA sequence in order to identify the gene structure (http://gsds.cbi.pku.edu.cn/) [88]. Protein sequence motifs were predicted by the motif elicitation program (MEME, http://meme.nbcr.net/meme3/mme.html) [89]. To identify the motifs, the conserved motifs were further queried in the InterPro database (http://www.ebi.ac.uk/interpro) [90]. The protein isoelectric point and molecular weight of NtMADS-box were predicted by ProtParam tool (http://web.expasy.org/protparam/) [91].

### 4.3. RNA Extraction, cDNA Preparation and Gene Chip

Total RNA was extracted with the SuperPure Plantpoly RNA Kit (GeneAnswer, BeiJing, China). All RNA samples were treated with RNase-free DNase I (GeneAnswer, BeiJing, China) and analyzed for integrity on a Bioanalyzer 2100 (Agilent technologies, USA). About 33.3 ng total RNA was used for amplification with the Amplification Kit (Thermo Fisher Scientific, Waltham, Massachusetts, USA). A total of 5.5 μg of the amplified product was fragmented by uracil-DNA glycosylase and apurinic/apyrimidinic endonuclease 1 (Thermo Fisher Scientific, USA).

The fragmented cDNA was labeled by terminal deoxynucleotidyltransferase using the DNA labeling reagent (Thermo Fisher Scientific, Waltham, Massachusetts, USA), which was covalently linked to biotin. The resulting labeled cDNAs (5.2 µg) were dissolved in 160 μL of hybridization mix solution, then denatured at 99 °C for 5 min. The mixed hybridization buffer was loaded into a microarray, and then the both septa were covered by round labels to prevent leaks and evaporation. An Affymetrix custom Tobacco Genome Array with feature Size 5 micron was used. A total of 80,652 tobacco genes were covered within this array. Tobacco *L25*, *EF1-alpha*, *Ntubc2*, *PP2A* genes were used as housekeeping genes. The RMA method provided by the R package, affy package, was used to conduct background correction, normalization, probe-specific background correction, probe summarization, and to convert probe level data to expression values. The hybridizations were performed in a hybridization oven (Thermo Fisher Scientific, Waltham, Massachusetts, USA) at 45 °C for 16 h. After hybridization, microarrays were washed by Fluidics Station 450 with wash buffer A and B (Thermo Fisher Scientific, USA). Three biological replicates were used in the Microarrays assay. Using the GeneChip Suite 5.0 default parameters, the detection *p*-value and the signal value were calculated for each probe set from each independent sample hybridization. The analyzed *p*-value was used to determine whether a transcript was reliably detected (present, *p*-value < 0.04). The data were further passed through a quality filtration using Microsoft Excel, according to the following criteria: the transcript must have been significantly expressed in at least two samples obtained under the same conditions (P for present, detection *p*-value < 0.04), and the median values of significant expression from replicate samples were used. The expression heat map was performed by R with the ggplot2 and pheatmap package.

### 4.4. Chromosomal Location and Gene Duplication

To determine the chromosomal locations of tobacco MADS-box genes, we obtained the physical genome annotation files from the China tobacco genome database V2.0. The interaction network was conducted by Circos software using the search of multiple proteins sequences [92]. The ORFs of gene pairs were aligned by the mafft program [93]. The synonymous substitution (Ks) and non-synonymous substitution (Ka) rates were calculated using the KaKs_calculator with default parameters [94].

### 4.5. Quantitative Real-Time PCR of Selected NtMADS-Box Genes

A total of 2 μg of total RNA in a 20 μL reaction was converted to cDNA with a SuperScript III Reverse Transcriptase (Invitrogen, Waltham, Massachusetts, USA) by the manufacturer’s instructions on an Eppendorf Mastercycler thermocycler (Eppendorf AG, Germany) with the following conditions: 25 °C for 5 min, 50 °C for 60 min, 70 °C for 15 min, followed by a hold at 4 °C until use in a qPCR reaction. A total of 60 μL of deionized water was added into 20 μL cDNA, and 1 μL of diluted cDNA mixture was used as the input for the qPCR reaction. qPCR reactions were made with a SuperReal PreMix Plus SYBR Green Kit (TIANGEN Biotech, BeiJing, China) following manufacturer’s instructions in a 20 μL volume. qPCR was done on an Applied Biosystems™ QuantStudio™ 6 Flex Real-Time PCR System (ThemoFisher Scientific, Waltham, Massachusetts, USA) with the following cycling conditions: 95 °C for 15 min, followed by 40 cycles of 95 °C for 10 s, 60 °C for 20 s, and 72 °C for 32 s. The melt curve conditions were 95 °C for 15 s, 60 °C for 1 min, 95 °C for 15 sec. All samples had only one melt temperature peak. The log2fold change was calculated by the 2^-ΔΔCT^ method using *26S* as a reference gene. The CT values represent the average of three technical replicates. The sequences of primers used for RT-qPCR are listed in Supplementary Table S4.

### 4.6. Plasmid Construction and Tobacco Transgenic Plant

Total RNA was purified from tobacco leaf and cDNA was obtained with the kit (Qiagen, Hilden, Germany). The full-length sequences of *NtMADS133* CDS were amplified with two primers. The CDS sequences were cloned into pDONR-zeo vector by BP reaction (Invitrogen, USA) and then cloned into pB2GW7 by LR reaction (Invitrogen, Waltham, Massachusetts, USA). The pB2GW7 containing the *NtMADS133* gene was transformed into tobacco leaves via Agrobacteria.

## 5. Conclusions

The pivotal role of the MADS-box gene family in plant growth and development has been well characterized in many plant species, however, information on MADS-box gene family in tobacco is still missing. Here, we conducted a genome-wide identification and expression analysis of the MADS-box family in *N. tabacum*. A total of 168 MADS-box genes were identified in the genome of *N. tabacum*. Phylogenetic and gene structure analysis revealed that NtMADS-box can be divided into two types, type I and type II, and clustered into four sub-families of Mα, Mγ, MIKC*, and MIKC^C^. Microarray-based analysis of NtMADS-box gene expression profiles in tissues at different developmental stages revealed that members of different NtMADS-box gene subfamilies might play specific roles in the growth and flower development of tobacco. Moreover, the expression patterns of selected NtMADS-box genes were further confirmed by qPCR. Importantly, overexpressed *NtSOC1*/*NtMADS133* could promote early flowering and the dwarfism phenotype in the transgenic tobacco plants, suggesting their conserved role in the development of growth and flowers in planta. Taken together, our findings provide insights on the characterization of NtMADS-box genes to further study their functions in plant development.

## Data Availability

The original data that support the findings of this study are available from National Tobacco Gene Research Centre at Zhengzhou Tobacco Research Institute but restrictions apply to the availability of these data, which were used under license for the current study, and so are not publicly available. Data are however available from the authors upon reasonable request and with permission of National Tobacco Gene Research Centre at Zhengzhou Tobacco Research Institute.

## Supplementary Materials

The following are available online at https://www.mdpi.com/1422-0067/20/20/5043/s1.
