# Supplementary material for: Genome-Wide Identification, Gene Structure and Expression Analysis of the MADS-Box Gene Family Indicate Their Function in the Development of Tobacco (Nicotiana tabacum L.)
Source: Int J Mol Sci. 2019 Oct 11;20(20):5043. doi: 10.3390/ijms20205043 (PMC6829366; doi:10.3390/ijms20205043)
Supplement: Supplementary file 1 [file ijms-20-05043-s001.zip › ijms-560114-SI-R2-revised/Supplemental Figure 1.pptx]

## Slide 1
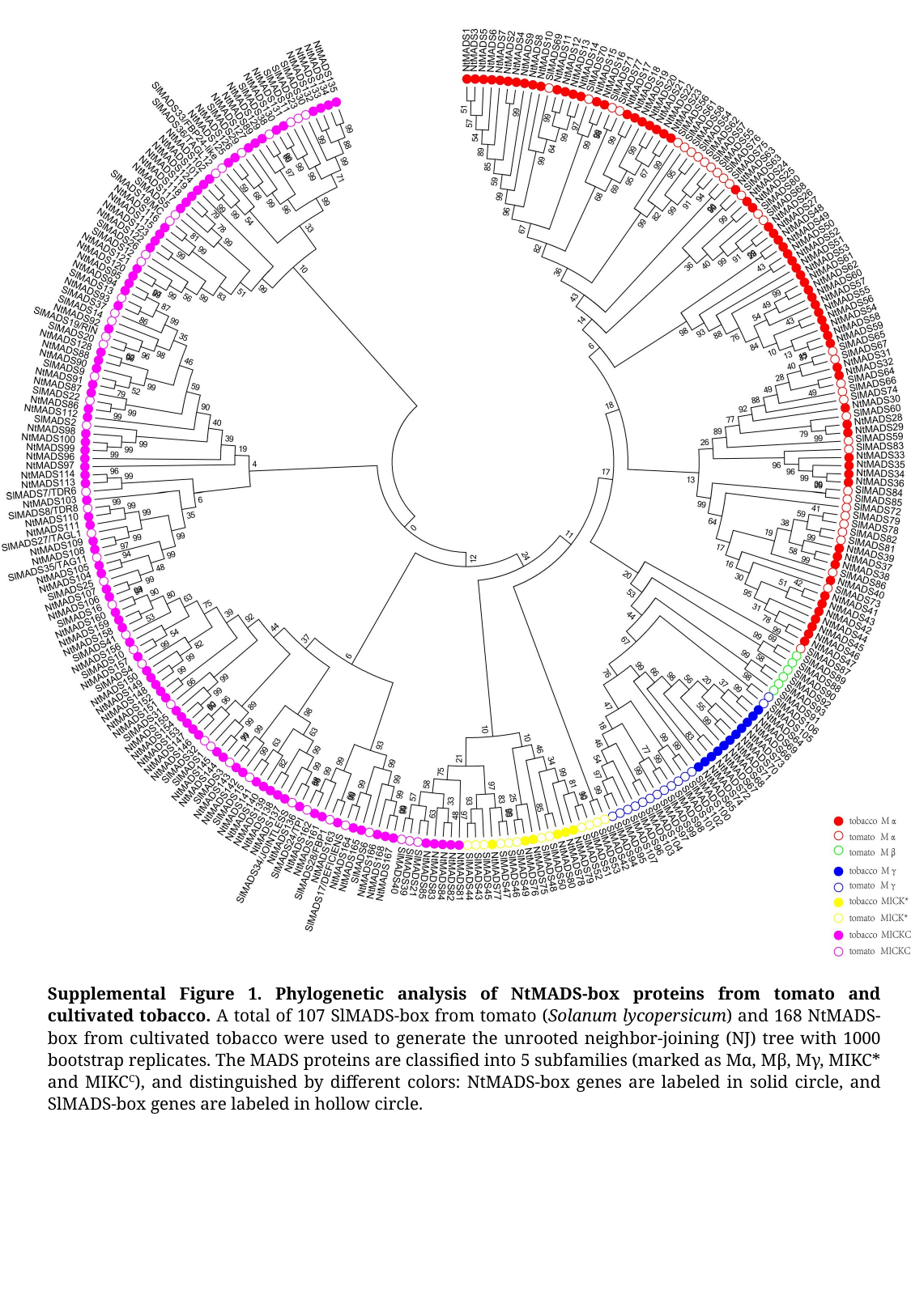

Supplemental Figure 1. Phylogenetic analysis of NtMADS-box proteins from tomato and cultivated tobacco. A total of 107 SlMADS-box from tomato (Solanum lycopersicum) and 168 NtMADS-box from cultivated tobacco were used to generate the unrooted neighbor-joining (NJ) tree with 1000 bootstrap replicates. The MADS proteins are classified into 5 subfamilies (marked as Mα, Mβ, Mγ, MIKC* and MIKCC), and distinguished by different colors: NtMADS-box genes are labeled in solid circle, and SlMADS-box genes are labeled in hollow circle.
